# What it means to thrive: a qualitative, interdisciplinary exploration of employees’ understandings of “thriving” at work

**DOI:** 10.1080/17482631.2025.2593081

**Published:** 2025-11-30

**Authors:** Cristen Dalessandro, Alexander Lovell

**Affiliations:** a O.C. Tanner Institute, 1930 S State St., UT, Salt Lake City, USA

**Keywords:** Thriving, workplace, employees, focus groups, human resources

## Abstract

**Purpose:**

Due to widespread societal changes, scholars and human resources (HR) practitioners need additional ideas and strategies to encourage employee well-being and “thriving” in the post-COVID workplace. However, academics and practitioners are typically not in conversation on this topic, which can lead to gaps between knowledge and practice.

**Methods:**

Informed by an interdisciplinary approach that bridges theoretical insights from psychology, sociology, and HR practitioner-focused research, we use focus group interviews with 113 full-time employees across five U.S. cities to explore how employees define what it means to thrive at work in contemporary times.

**Results:**

We find that employees’ accounts demonstrate the importance of both psychological and interactional factors in their understandings of thriving, including feeling a sense of personal well-being and opportunity at work, feeling appreciated in their interactions with coworkers and leaders, and feeling that the organization has a larger purpose and mission and that they, as employees, contribute to the organization’s purpose and success.

**Conclusion:**

Given employees’ interpretations, our findings suggest concrete strategies that HR practitioners can take to help employees thrive in the workplace today.

## Introduction

In the last six years since the beginning of the COVID−19 pandemic, immense social and economic shifts have made many employees across the world feel that change is the only constant in their work lives (Lovell et al., 2023). Related to this ongoing uncertainty, employee morale has suffered. Stress and anxiety have risen for workers across the globe, and as change and uncertainty will likely continue to be a characteristic of work in the immediate future, poor mental health outcomes may worsen (Crayne, 2020; Dalessandro & Lovell, 2023). Indeed, so many aspects of work have been upended in the last six years that researchers argue work will never be the same (Chafi et al., 2022) and a great deal of pre−2020 research on workplace-related subjects should be updated (Torres & Orhan, 2023). Considering these developments, human resources (HR) professionals need additional ideas and strategies to foster employee well-being and thriving in workplace contexts (Collings et al., 2021).

Organizational psychology typically treats “thriving” at work as a psychological state that leads to beneficial employee outcomes (Jiang, 2017). For example, research has found that thriving employees are more adaptable, more creative, more supportive of their coworkers, and less burned out compared to those who don’t believe they are thriving (Jiang, 2017; Kleine et al., 2019; Yang et al., 2021). Employee thriving also yields organizational benefits. For instance, thriving employees tend to be more promotive of, and committed to, their organizations (Kleine et al., 2019). Yet in post-COVID times, questions remain regarding how employees themselves define what it means to thrive at work today and how HR professionals might help employees reach a state of thriving.

Existing research, such as Spreitzer and colleagues’ (2005) pivotal work on thriving, takes a largely psychological approach. However, we need more information on the potential significance of social interactions (with both coworkers and the workplace environment) in shaping employees’ sense of thriving. Further, while many studies have explored the concept of thriving using quantitative data, qualitative research on this subject is much less common (for exceptions, see Niessen et al., 2012; Spreitzer et al., 2012). This is notable, as qualitative methods are well-suited to capturing complex meanings and concepts. In addition, employees’ experiences of the workplace have fundamentally changed since 2020 due to the reshuffling of workplace norms post-COVID (Gershon, 2024). What does thriving mean to employees today, and how might HR professionals best strategize to help employees thrive?

To answer these questions, we use data from 15 focus group interviews with 113 employees working full-time in the United States to explore, from an employee point of view, what it means to thrive at work. We take a unique interdisciplinary approach that bridges insights from the fields of psychology (self-determination theory), sociology (symbolic interactionism), and HR industry research (the “Talent Magnets” framework of workplace culture). Our results reveal that employees draw from personal and interactional experiences to help them determine whether they are thriving in the workplace. Using a qualitative approach, our research provides insight into the question of how employees themselves understand the concept of thriving in contemporary times. In addition, our results reveal practical tips that HR practitioners can use to create a workplace environment where employees thrive.

## Review of the Literature

Social science research has typically treated the concept of “thriving” at work as a psychological state in which employees feel a sense of “vitality” as well as a sense of learning or growth (Rawat & Varghese, 2018; Spreitzer et al., 2005). At the same time, scholars have acknowledged that the workplace context is important (Shahid et al., 2021; Walumbwa et al., 2018). Below, we review the approaches of several fields (psychology, sociology, and HR practitioner research) to explore what each might contribute to our understanding of an updated approach to thriving at work.

### Psychological research and thriving at work

Compared to other fields, psychologists have arguably been the most active when it comes to engaging with the issue of thriving at work (Liang et al., 2024). Much of the discussion on thriving, then, has focused on the individual. For example, organizational psychologists—who have contributed to much of the extant research on employee thriving—have most often treated thriving in a workplace context as a psychological state in which workers feel a sense of personal growth and development through their careers (Jiang, 2017; Porath et al., 2012; Spreitzer and Porath 2014). Psychological theories of thriving in the workplace argue that thriving positively impacts employees’ actions. For instance, workers who feel they are thriving are more likely to be engaged, pursue learning experiences actively, and be more effective at work in general (Jiang, 2017; Porath & Bateman, 2006; Porath et al., 2012; Spreitzer & Porath 2014).

Though theories of thriving in the workplace emphasize that thriving is an individual characteristic, organizational psychologists also acknowledge the critical importance of the workplace environment in supporting—or constraining—thriving (Jiang et al., 2019; Nawaz et al., 2020). The relationship between individual thriving and the workplace environment is also cyclical—while the workplace can impact employees’ sense of thriving, it’s also the case that when employees themselves feel they are thriving, their actions help shape the workplace into an environment that is supportive of thriving (Jiang et al., 2019; Tolentino et al., 2014). This view aligns closely with the tenets of self-determination theory (SDT), which some researchers have used to understand the psychological concept of thriving inside and outside of work.

According to Spreitzer and Porath (2014), self-determination theory (SDT) posits that all humans desire psychological growth and development, or a desire to thrive psychologically. Yet simultaneously, larger social contexts (such as the workplace) can encourage—or thwart—individual thriving. The fulfillment of certain psychological needs (autonomy, competency, and relatedness) is a key avenue through which individual growth, development, and well-being is achieved. Although workplaces can support the fulfillment of these needs, at times, they can also discourage them (Baumeister & Leary, 1995; deCharms, 1968; Harter, 1978; Ryan, 1995; Ryan & Deci 2000; White, 1963). Perhaps unsurprisingly, individuals’ well-being is negatively impacted when these needs go unfulfilled (Ryan & Deci, 2019). Though the terminology differs slightly, many researchers have treated the SDT’s approach to “wellness” as a measurement of whether or not individuals are thriving in their environment (Šakan et al., 2020). Although psychological research on thriving has highlighted the importance of environmental contexts in determining individuals’ thriving (Shahid et al., 2021; Spreitzer et al., 2005; Walumbwa et al., 2018), most psychological research centers the individual. Thus, we argue that insight from the field of sociology can add nuance when it comes to exploring how employees think about thriving at work in contemporary times.

### Sociological research and thriving at work

Though research on work and occupations is a large subfield within sociology, sociologists have rarely published on the concept of thriving in a way comparable to psychologists (Blankenship, 2010). However, this does not mean sociologists are uninterested in thriving. Instead, sociologists are very concerned with well-being—which is related to, but technically distinct from, thriving itself (Spreitzer et al., 2005). Sociological investigations of thriving typically explore the concept differently and with more field-specific terminology. For example, sociologists studying the workplace in contemporary times are often concerned with how the workplace experience impacts individuals’ mental and physical health outcomes differently across identity lines (such as race, social class, and gender) (Brick et al., 2023; Gevaert et al., 2020; Lee et al., 2019). Thus, while sociology is concerned with individual outcomes, it is also concerned with determining how individuals’ membership in different social groups can impact their well-being. Alternatively, some sociological research using the term “thriving” deploys the term in ways inconsistent with psychological research—for example, to describe the adaptability of organizations rather than individuals (Brint et al., 2016).

What sociology and psychology share is the acknowledgment that individual thriving outcomes are linked to the social environment. Yet, the two fields have different theoretical approaches to this idea. Sociologists tend to concentrate more heavily on the significance of the social environment for determining individual understandings of the world—a view commonly espoused, for example, by scholars of symbolic interactionism. According to the interactionist perspective, individuals learn about themselves and situate themselves in their social worlds via interactions with others and the world around them (Anthony & McCabe, 2015; Schwalbe, 1996). For instance, one concern of interactionist scholars is how individuals give meaning to their identities based on cues they receive from interacting with their environments (including the people in those environments) (Denzin, 2004). As individuals actively co-create their realities through interaction, this cycle becomes self-sustaining. Over time, socially agreed-upon definitions and ideas (such as what it means to thrive, for example) can—and typically do—shift and change (Berger & Luckmann, 1967; Goh et al., 2022; Ridgeway, 2011). Thus, while the psychological need to feel a sense of well-being may remain a consistent aspect of the human experience, explicit understandings of *how* to reach that sense of well-being in a workplace context can shift. Therefore, it is worth investigating how employees define thriving today given the momentous shifts in the workplace over the last few years.

At the same time, sociological research has generally not explored from an interactionist perspective how individuals come to understand what it means to thrive at work and how workers situate themselves as thriving (or not) based on those definitions. Thus, sociologists have generally not reached audiences concerned with what might be done to help employees thrive at work. Instead, HR practitioners have undertaken their own studies to identify strategies practitioners might use to help employees thrive. While academic HR scholars have generally taken a more quantitative, psychological approach, perhaps due to their responsibility for cultivating environments where large groups of employees can thrive, HR *practitioners* have taken a more holistic approach.

### HR Industry research on thriving at work

Recently, human resources academics have recognized a scholarly interest in the concept of thriving. For instance, Rice and colleagues’ recent work (2025) has confirmed that workplace factors such as employees’ interactions with leadership matter a great deal when it comes to employees’ sense of thriving at work. In addition, Wu et al. (2024) have found that “mindfulness” practices that keep employees focused on purpose also support feelings of thriving at work. In light of this increased research output, HR practitioners need insight into translating research into practical actions.

Due to HR professionals’ need for practical approaches to creating and supporting a thriving workforce, industry researchers at different consulting firms have developed action-oriented frameworks. One such model is the “Talent Magnets” framework, developed by researchers at the O.C. Tanner Institute (Utah, USA). We elected to investigate this framework here due to its emphasis on application as well as its current absence in the academic literature despite its use by HR practitioners.

O.C. Tanner is an employee recognition company that also offers culture consulting services, and researchers at the O.C. Tanner Institute focus on corporate culture and the employee experience. Although the Talent Magnets framework was developed as a consulting tool, it is closely related to the positive organizational scholarship turn in organizational studies. For instance, positive organizational scholarship research explores which factors explain and predict positive outcomes—including resilience, meaningfulness, feelings of connection, and so on—for individuals, groups, and organizations (Cameron et al. 2003). The development of the Talent Magnets framework is just one example of a consulting framework that aims to advise how to bring about positive outcomes in the workplace.

As a consulting tool, researchers developed the Talent Magnets framework using both qualitative and quantitative methods to uncover “what employees value most (and least) in a desirable workplace” (Lovell et al., 2022; Lovell et al., 2018). The Talent Magnets are so named because they “attract” people to certain workplaces and thus, result in more positive outcomes for both individuals and organizations. The six Magnets are as follows: leadership, opportunity, success, purpose, appreciation, and well-being (a visual representation of the Magnets can be found in the Appendix). “Leadership” considers employees’ experiences with their leaders. “Opportunity” considers whether employees feel they have room to develop and grow in their jobs. “Success” considers if an employee feels their organization is successful in their market, if their team is successful in their organization, and if they are successful within their team. “Purpose” considers whether employees feel they understand the organization’s purpose and how their role contributes. “Appreciation” considers whether employees feel appreciated for the work they do. Lastly, “well-being” refers to employees’ holistic sense of their mental, emotional, and physical well-being, and how work influences their well-being.

While the Talent Magnets framework is concerned with certain aspects of the individual employee experience, ultimately, improving each area is a matter of changing company culture (Lovell et al., 2018). Thus, similar to SDT and symbolic interaction, the Talent Magnet framework highlights the importance of connecting micro-psychological and environmental forces. The Talent Magnet framework also identifies “thriving” as an outcome of what happens when employees rate their organizations highly in each magnet area—employees and organizations both thrive together (albeit the use of the term “thriving” is aimed at a practitioner, rather than an academic, audience).

Yet, while HR practitioners have used the Talent Magnet framework to inform practices and policies in organizations, there is a lack of peer-reviewed academic research on this framework. Thus, while used in practitioner circles, academics have typically not engaged with the elements of the Talent Magnet framework holistically. However, academic research studies have separately established the importance of each “Magnet” for influencing positive employee and organizational outcomes. For instance, research has found that leadership styles are significant in influencing positive employee and organizational outcomes (Toor & Ofori, 2009). In addition, other research has found that employees need to feel appreciated and have a sense of opportunity and growth in order to feel highly engaged at work (Gulyani & Sharma, 2018). Some research has made explicit connections between “thriving” and Talent Magnet characteristics—such as work by Walumbwa and colleagues (2018), which found connections between employees’ perceptions of organizational thriving (including whether or not organizations were successful and had a strong collective purpose) and individual well-being. Last but not least, concerning well-being, a number of studies have found strong links between employee well-being and positive organizational outcomes (Dalessandro & Lovell, 2023). In sum, while the Talent Magnet framework itself has not received attention outside of consulting and practitioner circles, existing academic research suggests that there may be a relationship between the Magnets and employee definitions of thriving.

Though potentially helpful as a tool, from an academic perspective we must ask: in a post-COVID workplace context, do the Talent Magnets still come to mind when employees are asked to interpret what it means to thrive at work today? Further, the Talent Magnets framework—due to being practitioner-focused—has not been comprehensively tested through the lens of psychological and sociological theories regarding thriving and the employee experience. Thus, the results below aim to identify how employees make sense of the concept of thriving at the individual and interactional levels and how closely the Talent Magnets framework maps onto employees’ accounts. This investigation helps bridge interdisciplinary academic and academic-industry gaps and opens the conversation for building solutions to help more employees thrive in the workplace.

## Materials and methods

We collected the data used here as part of a larger project on employees' workplace experiences— specifically, their experiences with different workplace trends (such as increased flexibility) since 2020. The data are composed of both qualitative interviews and a quantitative survey. However, here we focus solely on the project's qualitative component to highlight how employees make sense of the concept of thriving. We worked with Pearl IRB (Indianapolis, IN, USA)—an independent IRB that serves both academic and non-academic institutions—to review, exempt, and approve different components of the multi-method studies and to confirm adherence to all ethical standards in research involving human subjects. The Pearl IRB found the study “exempt” and did not provide a case number since it did not advance to a full board review.

We included focus groups in the larger research project for a few reasons. First, despite the inclusion of qualitative research in certain fields (such as sociology, for example), using qualitative methods to explore employees’ experiences is surprisingly scarce in other fields (such as academic HR and psychology research). At the same time, we needed to conduct qualitative interview research to get a fuller picture of how employees define thriving—something that would be extremely difficult to gather using quantitative methods. Thus, we chose to use focus groups to best capture how employees are defining thriving through interaction with each other.

To represent a wide range of regional U.S. perspectives, the focus groups took place in person across several U.S. cities—Chicago, IL; New York, NY; Nashville, TN; Minneapolis, MN; and Los Angeles, CA in the winter of 2022−2023. Thus, the views expressed by participants best represent those of employees working in the United States. Although these five cities are not representative of all workers in the United States, they provide a mix of perspectives from different regions of the country, including the East Coast (New York), the West Coast (Los Angeles), the Midwest (Chicago and Minneapolis), and the South (Nashville). In addition, each of the chosen cities is made up of diverse groups of workers employed across a range of industries.

We elected to conduct focus groups as opposed to one-to-one interviews in order to capture employees’ interactions with each other on the topic of thriving at work. We conducted a total of 15 groups (three groups per city), which varied from five to nine participants in each of the groups. Although researchers have not agreed when it comes to *either* how many focus groups *or* how many participants per focus group are ideal, best estimates posit that more than four groups are needed for data saturation and that the ideal number of participants per group is no more than 10 (Hennick & Kaiser, 2019; Krueger & Casey, 2008). We recruited 10 participants with the hopes of seating eight per group, although some groups had fewer than eight participants due to no-shows. Although we began to reach data saturation before completing all of the interviews, we did not cancel any interviews to account for the possibility of seeing variations between the different cities/regions. However, upon completing interviews in the five cities, we determined that we did indeed reach data saturation and that we did not need any additional interviews.

The research team recruited for the project with the assistance of a marketing research firm that operates globally in key markets to help connect researchers with study participants. This firm helped recruit participants but did not participate in study design, data collection, analysis, or data interpretation and was limited to helping the research team access participants based on pre-defined criteria determined by the research team. Via a guide developed by the authors, the research firm screened potential participants for eligibility and issued incentives ranging from $125 to $185 (depending on location and difficulty recruiting subjects). We assessed that these payments were high enough to incentivize participants to attend and participate in interviews, but not high enough to unfairly influence results.

To be considered for a focus group, potential participants had to work full-time, answer an articulation question (share their favorite historical figure, articulating why), and agree to attend the 90-minute focus group on the interview date. We asked individuals to complete an articulation question in the hopes of finding participants who would be willing to share and explain their answers to our questions in a group setting. All focus groups took place at the local offices of the marketing research firm, which are centrally located in each city under study.

With the firm's help, we recruited a diverse sample in terms of gender, racial/ethnic identity, education, access to technology at work (regular access versus limited access), and job roles (both blue-collar and white-collar roles). The authors conducted three focus groups at each research site: one group of self-identified women, one group of self-identified men, and one mixed-gender group. Ultimately, 62 women and 51 men participated in the groups. The racial/ethnic breakdown of participants was as follows: 49 self-identified as white, 35 as Black, 12 as Asian, 11 as Hispanic, and six as some other racial/ethnic identity. In addition, the ages of participants ranged from 26 to 59, with 53 participants being in the 20−39 age range and 60 being in the 40−59 age range. We also recruited employees working in a range of industries, including (for example) manufacturing, healthcare, finance, transportation, sales, technology, and retail. These employees also worked in a range of job blue- and white-collar roles, including (but not limited to) consulting, management, sales associates, engineers, analysts, and assembly line workers. Due to the diversity of our sample (including geographic diversity), we avoided over-representing any groups in the research.

Before each focus group, participants signed an informed consent form. The authors took turns moderating the focus groups (one moderator per group). We recorded all interviews for transcription and took the steps necessary to protect the data and identifiable information to the extent possible with focus groups. Any identifiable information in our results below has been changed to protect participant confidentiality. We also limited information on participants’ specific work roles below (unless that information was pertinent to the example) to protect confidentiality as best as possible. All names of participants are pseudonyms.

The authors developed the interview guide for the focus groups. All topics covered concerned various aspects of the employee experience. Specifically, in alignment with the topical goals of the larger project, we focused heavily (though not exclusively) on employee experiences with workplace flexibility, learning and development opportunities, and adapting to changes in the workplace since the beginning of the COVID−19 pandemic. All groups additionally covered the meaning of thriving in a workplace context, asking employees, “What does it mean to thrive at work?” We asked this question due to our research team’s interest in the topic and our understanding that, with few exceptions (Gielissen et al., 2021; Hyman & Doolittle, 2022), qualitative research on thriving at work is uncommon. Answers to, and conversations around, this question serve as the principal source of data for this paper.

Our analysis utilizes a modified grounded theory approach (Charmaz, 2014; James-Hawkins et al., 2019) since while we allowed participants to discuss thriving in an open-ended fashion, we did go into our research project with one of our goals being to identify what it means to thrive. We asked our participants specifically what it means to thrive, but we then allowed them to expand upon different relevant threads that came up in conversation. Our interviews were thus open-ended—we only prompted participants to tell us what “thriving at work” means to them. We did not ask participants questions about any of the Talent Magnets; instead, we allowed any linkages to arise organically in conversation.

We conducted coding and analysis simultaneously (see Charmaz, 2014) and approached coding specifically using the guidelines set by Braun and Clarke (2006)—a “thematic analysis” approach. We began by first reading each interview to identify broad patterns in the data. For this analysis, we focused specifically on responses to the “what it means to thrive” prompt (as detailed above), which therefore involved concentrating on a specific part of the transcripts for coding. In doing so, we identified that employees’ definitions of what it means to thrive fell broadly into two camps: more personalized meanings/definitions of thriving and definitions connected to larger contextual factors (such as whether the organization was “thriving”).

Upon discussing these two patterns (personal and contextual approaches to thriving), we further identified that participants’ approaches could be characterized as psychological or interactional, with “interactional” factors further divided into either interactions with coworkers/individuals and interactions with the general cultural environment of the workplace. We then coded for responses fitting into these broad themes and later, interpreted how each of the themes lined up with the Talent Magnet definitions. Our associations of the themes with each Magnet were based on the definitions of the Magnets outlined in previous research (discussed above) (Lovell et al., 2018). We addressed intercoder reliability in several ways, as outlined by Cofie and colleagues (2022). For instance, both authors working on the project had prior experience with qualitative work and had ongoing discussions about the data with each other throughout the analysis process. While the first author completed much of the coding, the second author also read through the transcripts and confirmed the validity of the codes used in the process. Keeping one author further removed from, yet still actively involved with, the coding process helps correct for biases that could arise (Cofie et al., 2022).

## Results

### Psychological factors—a sense of personal well-being and opportunity

The first set of criteria by which employees defined “thriving” in focus groups centered on what could be considered personal psychological factors. These are concerns that, while linked to individual employees’ environments and social interactions, are ultimately about what employees’ experiences in their workplace do for them psychologically and how those experiences fulfill the needs of autonomy, competency, and relatedness. In discussions, employees made sense of themselves as thriving when they felt a sense of mental and physical well-being and when they felt they had ample opportunities to grow and develop as individuals at work.

For many employees, thinking about “thriving” at work made them consider how work made them feel personally. Feeling personal sentiments of “enjoyment,” “balance,” “fulfillment,” and a sense that work was “rewarding” were terms that arose time and again in discussions linking workplace thriving to personal well-being. For example, Angela (Los Angeles) shared, “I feel like thriving for me is first and foremost a mental thing. When you get up in the morning and you *want* to be there, and not [feel] you *have* to be there.” Similarly, Lawrence (Nashville) shared, “[Thriving is] looking forward to going into work. If you’re enjoying your work and thriving at it, you look forward to the next day. I can’t always say that about some places that I’ve worked at in the past!” For Angela and Lawrence, well-being is about their psychological response to work, and whether those responses make them feel they are enjoying their time there.

Other participants made connections between “thriving” and the fulfillment of the psychological needs of autonomy, competency, and relatedness. For example, Leanne (Nashville) shared,

*For me personally, [to be] thriving, I’m hitting those numbers. The team is, everybody’s on one page and it’s just flowing, we’re cohesive, we’re a unit. I’m not stressed, I love what I do, and I’m thriving under stress and high pressure. The energy’s flowing, there are days you look up [and say], “oh it’s four o’clock already!” I go home and then I don’t take the job with me. But to me that’s thriving. It makes me want to—[the job] makes me love what I do.*

Leanne’s quote demonstrates the importance of feeling a sense of autonomy and competency at work and the importance of feeling a sense of connection and cohesiveness with colleagues. Her narrative constructs thriving as something that, while connected to social processes, ultimately is assessed by how it makes her psychologically feel at the end of the day (work that she loves and that promotes a healthy work-life balance). As we discuss more below, her quote also reflects the importance of having a collective purpose with her team in order to realize a personal sense of thriving.

In addition to a general sense of well-being, many participants made connections between positive psychological states and having a sense of opportunity at work. Participants spoke of reaching goals, opportunities for growth and development, and having a career path or plan as crucial to their own personal sense of thriving. Though some spoke of being promoted, participants also thought of thriving in terms of opportunities for growth and development in their current roles. For example, Marissa (Los Angeles) shared,

*I would say thriving at work for me would mean I feel accomplished. I’m getting ahead, meaning I’m learning new things. I’m doing new things, becoming more of an asset [to] the company, and just really feel good about what I’ve done, whether it’s a department or for the company. That’s what I feel is the definition for thriving.*

Though organizational culture matters for whether or not she feels a sense of opportunity, Marissa’s quote makes clear that opportunity is also a personal matter with psychological implications. Similarly, Lori (from the same Los Angeles focus group) added, “[Thriving is when]…you’ve totally mastered what you're doing. You're ready for another accomplishment, another something that makes you keep building on yourself.” These quotes demonstrate the importance of a sense of opportunity for fulfilling the psychological needs of autonomy, competency, and relatedness. However, this quote from Henry (from another Los Angeles group) also illustrates how universal these desires can be:

*I think everybody here in the world is looking for the same thing. We want to feel that we are contributing something worthwhile. And in many jobs, you don't feel that way. I think as long as you feel like you're doing something, [doing] some good, yes. That's what everybody's looking for.*

As Henry observed, the desire to contribute “something worthwhile” is personal, but also shared.

What these quotes demonstrate is that one way participants interpret what “thriving” means is at the personal and psychological level. These factors map closely to the “well-being” and “opportunity” Talent Magnets and directly speak to the fulfillment of the psychological needs of autonomy, competency, and relatedness. At the same time, as the quotes above suggest, social interactions with coworkers and with one’s environment are vital in determining whether employees’ psychological needs are met. Employees do not just understand “thriving” as the fulfillment of individual psychological needs. Through interpersonal interactions at work, they also come to understand what thriving means and how thriving is achieved.

### Interacting with coworkers—appreciation and leadership

The messages employees receive via their interactions with their coworkers and leaders make a difference in their assessments of their own thriving. In interpreting these messages, employees come to understand what it means to thrive and whether they are thriving at work. For instance, employees said they come to understand themselves as thriving when they feel appreciated and acknowledged (by both leaders and other coworkers) for their efforts. In addition, leaders help set the tone for whether the work environment is a space where employees can thrive. Leaders’ attitudes and approaches send messages to employees about whether they, as individuals, are thriving.

Expressions of appreciation from coworkers and leaders are essential in defining not just a personal sense of thriving but also a sense that an individual is thriving in the eyes of their peers (including leaders) within the organization. For example, Magda (New York) shared, “I think getting good feedback is thriving at work as well, whether from your coworkers or your management…Constructive criticism definitely from my coworkers and from my supervisors [helps] to just grow, to be better at my job, to be open to learning more things.” In Magda’s case, feedback and “constructive criticism” helped her understand what it would take to thrive at work and help her understand what, if anything, she would need to do to improve at her job.

*Christian’s (Los Angeles) perspective illustrates this point even further: I think a couple of people [in the group] have mentioned goals….And if on top of that your manager or teammates recognize you then that's another indicator that you're doing well. And if on top of that the organization recognizes you then that’s like a triple crown. I think those are three indicators to help you know you're doing well.*

For Christian, in addition to meeting one’s personal goals (which reflects the sense of “opportunity” mentioned in the last section), feeling a sense of appreciation from coworkers, leaders, and the organization is an indicator for thriving. In both examples, interactions with coworkers also helped Magda and Christian get closer toward fulfilling the needs of autonomy and competency by helping them feel they are valued workers.

Other participants voiced similar sentiments. For example, Evan from Nashville shared, “I think humans need acknowledgment…I think it really encourages them when somebody else notices what they do…[hard work] means even more when other people see it, and they acknowledge it, too.” Similarly, Rosa (Los Angeles) said,

*I feel like [thriving is] when all of the employees feel positive about what they do and they’re also receiving some type of acknowledgment from upper management, as well as clients or customers giving you that type of feedback. [It's] just kind of an energy where you can feel it everywhere, anywhere you go.*

Based on these quotes, employees understand their own thriving to be partially contingent upon how other people associated with the organization are defining it. Acknowledgment from coworkers and management that they are on the right track helps individual employees understand whether they can consider themselves to be thriving (or not) at work. If employees believe they live up to expectations—expectations that are established through their social interactions—their psychological needs are also fulfilled, and they understand themselves to be thriving.

At the same time, employees emphasized interactions with a specific social group in the workplace: leaders. Brad (Minneapolis) illustrates this with his perspective: “I’ve always felt like I was thriving when management treats everyone like human beings or like adults, doesn’t talk to down to them [and] acknowledges, ‘Hey, we’re going to do this job and we’ll trust you to do your job.’ [There's] no hovering, things like that.” Arguably more so than other employees, leaders have the power to influence whether employees feel they are thriving at work. For example, Adrian (Los Angeles) acknowledged that one of the managers in his workplace creates a poor work environment that makes it very difficult for employees to thrive:

*[He should] retire. He’s been doing it 50 years. He’s never going to change. People have complained, he’s made girls cry in the office. He’s not really a good person. I’ve had these arguments in the office about it, and he’s never going to change. People have called the main office, I know they’ve talked to him about it, but it just isn’t who he is. It’s not overall good for the store. That’s why we have a lot of problems with employees.*

Adrian’s interactions with this manager—in addition to his knowledge of other people’s interactions with him—led him to conclude that this manager’s behavior is negatively impacting the ability of employees to thrive. In this case, Adrian sees this manager as taking an outdated approach based on his interactions with the manager and his observations about how other people react to this individual. Based on coworkers’ disapproval and the negative emotions Adrian feels around this manager, he concludes that this individual should retire and make way for someone better suited to the environment who won’t get in the way of other employees’ attempts to thrive.

Overall, employees’ interactions with leaders and coworkers help them understand what it means to thrive—and to assess whether they are thriving—in their workplaces. Employee interactions also help them understand what a thriving workplace does and does not look like. These interactions—which involve assessing a sense of appreciation and leadership’s evaluations of them—can make them feel good about themselves and work. Employees thus believe themselves to be thriving and that their psychological needs are met. However, employees come to understand whether they are thriving (and whether their coworkers are thriving) largely through their interactions. Interactions that employees have with not only individuals in the work environment, but also the work environment more generally, matter for their understandings of thriving.

### Interacting with the organization—success and purpose

In addition to interactions with coworkers and their leaders, employees' social interactions with the workplace environment matter when it comes to having asense of thriving. These types of interactions closely match the “success” and “purpose” Talent Magnets for two reasons. First, these interactions reflect employees’ understandings of whether their organizations are invested in the success of not just senior leaders but all employees. Second, they indicate whether there is a collective purpose driving the entire organization forward. Participants expressed these sentiments throughout conversations about employees and organizations thriving in tandem or together. Employees take cues from their interactions with the organization as a whole (rather than just individual employees) to assess whether the organization is thriving and whether they are thriving in the organizational context. An organization’s reputation and a sense of its general cultural climate drive employees to understand what it means to thrive in the organizational context and whether organizational thriving also translates to the thriving of individual employees.

For example, Theo (Chicago) made connections between employees’ sense of thriving and the organizational environment:

*I think that if the company is doing well, or they're getting a lot of accolades or live press, that's going to help engagement and morale, possibly retention as well on something like that. It's not going to be a negative. I think the better the company does, the more people are going to want to engage [with] their workload…*

Theo’s quote illustrates how organizational and individual employee well-being can be intertwined. Similarly, Willow (Minneapolis), responding to another participant in her city who shared the idea that organizational and employee thriving is intertwined, said,

*I would agree with that, especially in my non-profit. The better they do, the better our [employees do], and word of mouth goes [around]. I think especially now with what we've been through with COVID, that employees had to make the company still survive. At least I hear all the time about restaurants—that people are going out of business and businesses are [failing].*

For Theo and Willow, a general sense that an organization is thriving has an impact on individual employee sense of thriving as well. Typically, a sense that the organization is thriving also encourages employees to think of themselves as thriving too, with one caveat: if employees perceive that their organizations are successful but they treat employees as disposable, it damages the sense of connection and relatedness needed to fulfill psychological needs. If this is the case, employees are less optimistic about their own personal thriving.

For instance, when employees feel that organizations are financially thriving but holding out on their employees, the idea that individuals are thriving is damaged. In one example, Amy (New York) shared, “Sometimes they [the organization] hold back, sometimes they keep all that wealth for themselves and they don't share it and it does not do anything for the little people like myself. It doesn't trickle down to the people who actually do the work and it does nothing for personal or professional growth.” Other employees voiced a similar sentiment—that organizations could make or break their relationship with employees depending on their willingness to share success. Leon’s (New York) quote illustrates this:

*Not to go off on a tangent, but I was thinking about this article that I read the other day. They were talking about employees silent quitting. Showing up to work, but you’re doing the bare minimum. The way to avoid that as an employer is to make sure that you're putting some respect on your employees because they are the ones making it possible for you to be getting all these numbers. When you can go out in front of the public and boast about your successes, we’re the ones doing the work. Make sure that we get respect.*

Leon’s quote demonstrates that if employees believe the organization does not value their contributions to success, it not only can be detrimental to individual employees, but it can also have a negative impact on employee willingness to continue supporting the organization with their time and energy.

Since having a sense that the organization is being unfair violates the psychological need of relatedness, employees understand this unfairness as hurting their own ability to thrive as individuals. Brenda (Nashville) voiced a similar sentiment:

*The job I came from, the company had their best two years that I was there, but we saw nothing for it. We had no raises, no bonuses. Kind of self-centered. It was privately held, so the man was getting richer and could build a bigger house, but nothing was being passed down. No kudos were being passed down to the people that made it happen. That’s why I quit. But I feel like where I work now, it’s definitely the opposite, at least from what I can see…It’s always something going on, recognition, their screen’s up saying who did what, and a lot of advancement as well.*

What these quotes show is the danger in *not* sharing success. If organizations claim to be healthy but employees don’t feel they are able to share in the success, they risk violating the psychological need of relatedness that employees need to thrive. At the same time, as Brenda’s quote illustrates, a general sense that success is shared leads individual employees to have confidence in their own sense of thriving.

In addition to messages of success, the ways in which organizations communicate messages of purpose matter for individual thriving. Feeling a sense of working toward advancing some purpose as a group could lead to an individual sense of thriving. For example, Talia (Minneapolis) shared,

*At least with the new company that I’m in…everybody’s so driven to bring in contracts whether you’re a director or you just got here out of college. Everybody’s like, let’s get these contracts, because we all benefit. I like that model… the leadership who’ve been there for a long time… a lot of them, they’re very open to if you have an ask or you see something, [they say] “Let us know, how can we fix it?” [They’ll say] “OK, let’s have a meeting, tell us your ideas and [we’ll] see what we can do.”*

For Talia, there is a strong sense of purpose at her organization that motivates employees to do their best work regardless of their tenure or rank. The willingness to work together is both cultural and has an impact on individual employees like her.

Marilyn (New York) also connected her own thriving to working together toward a shared purpose: “There's so many different groups that support [a project]…So I feel that I'm thriving when we're included. Because we're adding value. We’re brought to the meeting, because we're adding to the [project]. We're not just another [check] the box.” As Marilyn’s quote shows, when everyone is working toward a cohesive goal, it makes her feel like she is also thriving on a personal level. Tara (Los Angeles) points this out as well: “Yes, definitely the work culture…it’s everybody at the company feeling a sense of, we’re glad we’re here and we’re glad we’re doing it together, because without that, it’s miserable going to work.” Lastly, Rachel (Nashville) shared that striving toward a “common goal” is also important to her own work experiences: “[In] my line of work, I can’t do something without approval from my client. If I get stuck with something that I don’t know how to answer for them, there’s people on my team that I know I could reach out to. It’s just, I feel like everyone being on the same page and trying to meet that common goal.” What these quotes demonstrate is that a personal sense of thriving is connected to employees’ sense of working together with others and their organization as a whole to accomplish shared goals.

Feeling a sense of organizational success and purpose—two factors determined by the organizations where employees work—helps employees determine their own sense of thriving. In general, interviews revealed that employees see individual and organizational thriving as interconnected: how organizations treat employees in their own quest to thrive matters for individual employees’ own sense of thriving. However, when organizations’ messages violate the fulfillment of employees’ psychological needs (especially relatedness), a shared sense of thriving breaks down.

## Discussion

When it comes to thriving at work, more information is needed regarding how to define the concept from an employee perspective. Are employees’ understandings of thriving consistent with existing definitions? It is also important to understand how insights from diverse fields might help clarify employees’ understandings. For instance, Spreitzer and colleagues’ (2005) model of thriving defines it as a psychological state contingent upon feelings of “vitality” and “learning.” While useful, this emphasis on psychological or individual-level agency and adaptation does not expand upon the importance of social interactions with coworkers and the workplace environment in cultivating employees’ sense of thriving. In our work, we demonstrate the critical importance of employees’ interactions for informing their definitions of thriving, which encompass a set of experiences related to having a sense of well-being, purpose, opportunity, success, appreciation, and supportive leadership in the work environment that lead to positive self-assessments of thriving. Thus, while we found that thriving is a psychological state for employees, whether or not employees feel they are thriving (and how they define it) is contingent upon both psychological and social processes.

Informed by sociological and psychological insights along with the “Talent Magnet” framework (which is not generally utilized in extant research on the concept of thriving), our analysis has revealed the extent to which employees’ social interactions are significant for informing their understandings of what it means to thrive. Thus, regarding our main research question of interest (what does thriving mean to employees themselves?), we have found that employees understand thriving in ways consistent with the Talent Magnet framework and that rather than exist in isolation, employees’ understandings of thriving are also rooted in perceptions of whether the larger organization is thriving as well (see also Walumbwa et al., 2018). For example, employees develop a personal sense of thriving through gauging their feelings of well-being and opportunity at work. While a sense of well-being and opportunity speak to individuals’ psychological needs, employees’ assessments are also contingent upon whether their interactions with others and with the workplace environment foster and support well-being and opportunity. Employees also assess personal thriving by examining the appreciation and leadership they are exposed to at work—two factors that are highly contingent upon their social interactions as well. Lastly, employees understand their own thriving to be related to the organization's purpose and whether they feel the organization is set up for success.

Our results support the importance of context and leadership—rather than just individual psychological states in isolation—for individual employees’ assessments of thriving (see also Shahid et al., 2021; Walumbwa et al., 2018). In addition, the importance placed on well-being and opportunity are similar to the sense of “vitality” and “learning” captured by Spreitzer and colleagues (2005). However, ultimately, we find that thriving in contemporary times is multi-dimensional according to employees. Like Walumbwa and colleagues (2018), we find that collective thriving (for instance, at the organizational and team levels) also relates to individuals’ sense of their own thriving.

Our research has theoretical, methodological, and practical/managerial implications. First, regarding our theoretical contribution, our unique interdisciplinary approach merges theoretical insights from psychology, sociology, and human resources industry research. We find that rather than one perspective being most valuable, all three work together to inform our knowledge of how employees understand what it means to thrive at work. These employees’ accounts stress that fulfilling the psychological needs of autonomy, competency, and relatedness are important determinants of thriving. At the same time, employees decide whether those needs are met—and what it even means to thrive at work—through their social interactions with other individuals in the workplace and the workplace environment itself. In sum, employees’ mental processes combine with their interactions with coworkers and organizations to determine individual assessments of thriving.

At the same time, our research has shown that applying qualitative methods to this issue helps us zero in on how employees are making sense of the concept of thriving at work today. Our results demonstrate the importance of both mental and interactional factors for employees’ experiences at work as well as the potential utility of frameworks that align with positive organizational scholarship. Lastly, by including insights from HR industry research, we found that accounts from participants about what it means to thrive simultaneously map onto the “Talent Magnets” framework used by HR practitioners. Although academic fields sometimes do not speak to each other fully, they speak to industry or practitioner-focused research even less often. However, our work shows that academia and industry ideas can overlap in useful ways.

Regarding practical or managerial applications, as socially-agreed-upon definitions of what it means to thrive can change over time (see Berger & Luckmann, 1967; Goh et al., 2022; Ridgeway, 2011), it is important to determine the applicability of certain frameworks—such as the Talent Magnets—to the employee experience today. We found that this framework applies to the lives of those we interviewed, which opens the discussion for what HR practitioners can do to help employees thrive at their organizations. For instance, HR personnel can cultivate the Talent Magnets in their organizations by taking actions such as providing employees with ample opportunities in the workplace, implementing programs that support individual well-being, and training leaders to be engaged with those who report to them. Further, how organizations communicate their success and mission matters. If organizations put forth strong messages of success and purpose and demonstrate a shared commitment to valuing employees for their efforts, individual employees' psychological needs of autonomy, competency, and relatedness can be met, thus strengthening employees’ sense of thriving. However, if organizations do not act in ways that demonstrate that they value employees’ commitments, they violate the psychological need for relatedness, diminishing individual employees’ sense of thriving and thus seemingly weakening employee loyalty.

Although our research provides a much-needed perspective, it is not without limitations. First, we were only able to sample employees in the United States. Qualitative data from other countries is needed to add nuance to this issue, since definitions of thriving (and ways to achieve it) likely differ globally. Second, because our recruitment strategy targets major metropolitan areas, we are undoubtedly missing the perspectives of workers in more rural locales. In addition to geographic limitations, because this data is based on qualitative focus group interviews, our sample does not represent all workers in the United States, workers across all industries, or even all workers in the cities we sampled. Thus, future research directions include exploring the patterns we have identified using a more pointedly representative sample. Lastly, research has pointed out that even within HR, those who identify as HR “scholars” often differ greatly from those who identify as HR “practitioners” when it comes to their approaches to applying ideas to the field of human resources (Ren, 2017). Thus, because it was developed as a practitioner-focused consulting tool, the Talent Magnet framework is likely less known among scholars and, thus, there is currently not a body of academic research that tests the framework using different methodological approaches. However, as the call for scholar-practitioner collaboration continues to strengthen (Ren, 2017), hopefully this will be less of an issue in the future. Ultimately, despite our limitations, our research provides a much-needed qualitative perspective on the issue of what it means to thrive at work and opens up more questions that other researchers may wish to pursue with additional quantitative or qualitative data.

## Conclusion

In this paper, we aimed to explore how employees themselves define what it means to “thrive” in the workplace today. We found that employees’ interactions are critical for informing their understandings of what it means to thrive, and that the six “Talent Magnets” of purpose, opportunity, success, appreciation, well-being, and leadership can all be found in their accounts of making sense of thriving. Our interviews indicate that employees’ understandings of thriving inform their own sense of whether they are thriving or not as well as the point that workplace culture and context are critical in determining employees’ own personal assessments of their own thriving. HR practitioners can thus look to support one of more of the Talent Magnets in their organizations in order to encourage employee thriving in contemporary times.

## Supplementary Material

Supplementary Material Appendix Talent Magnets

## Data Availability

Due to our work with a private organization, the data are not publicly available. However, the data are available from the authors upon request.
