# Supplementary figures and images for: What it means to thrive: a qualitative, interdisciplinary exploration of employees’ understandings of “thriving” at work
Source: Int J Qual Stud Health Well-being. 2025 Nov 30;20(1):2593081. doi: 10.1080/17482631.2025.2593081 (PMC12671426; doi:10.1080/17482631.2025.2593081)

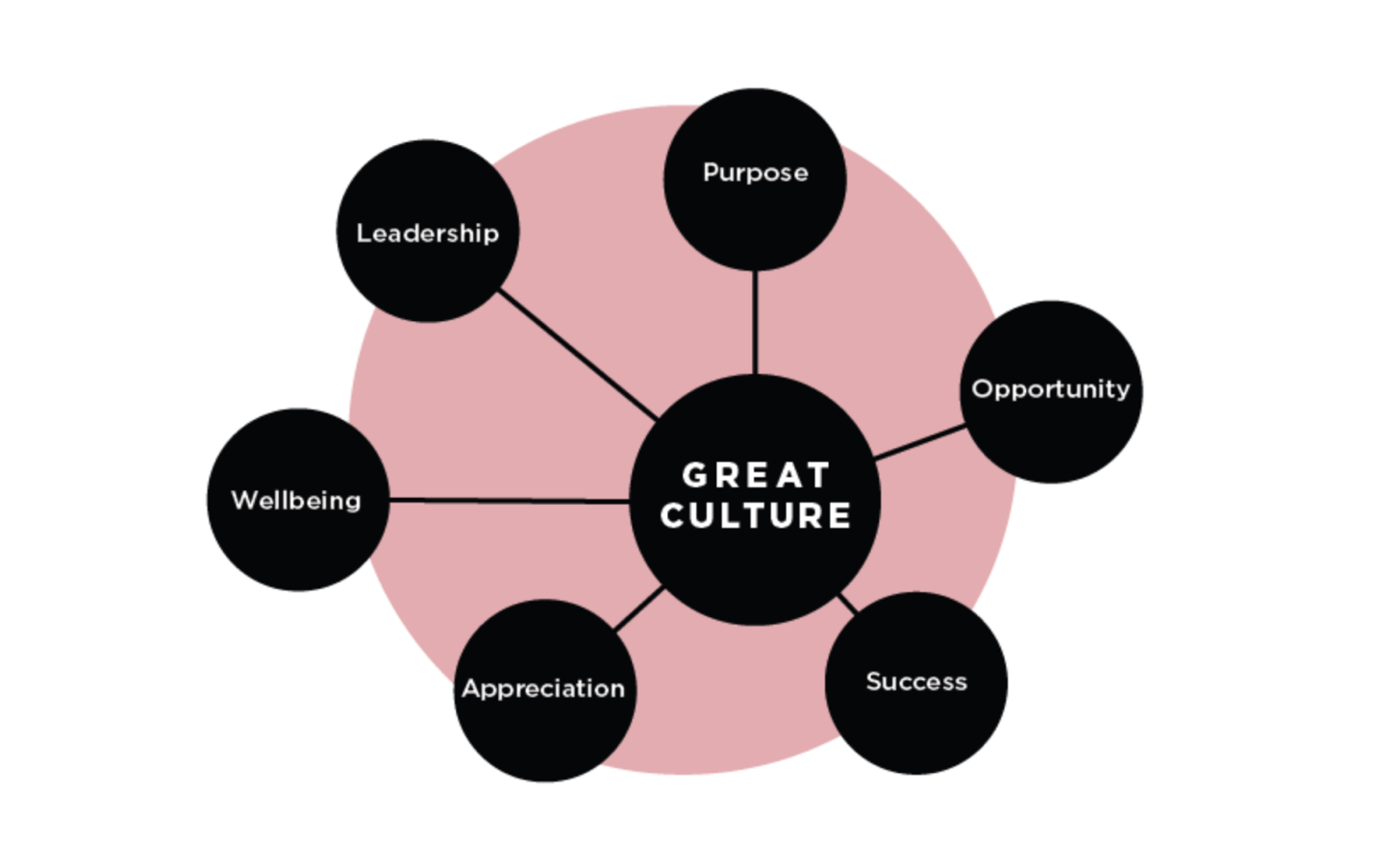


The Talent Magnet Framework

(Source: Lovell et al. 2018)

Supplement: Supplementary Material — Appendix Talent Magnets [file ZQHW_A_2593081_SM6653.docx]
